# Biopsia líquida: un reto para el laboratorio de diagnóstico clínico

**DOI:** 10.1515/almed-2020-0036

**Published:** 2020-08-28

**Authors:** Wladimiro Jiménez

**Affiliations:** Servicio de Bioquímica y Genética Molecular, Centro de Diagnóstico Biomédico, Hospital Clínico / Facultad de Medicina, Universidad de Barcelona, Barcelona, España

**Keywords:** ADN, biopsia líquida, next generation sequencing, PCR

En los últimos años el laboratorio de diagnóstico clínico ha afrontado numerosos retos de tipo científico, tecnológico y digital. Este fenómeno se ha asociado a una permanente actualización de los facultativos responsables de estos laboratorios. En este editorial pretendemos llamar la atención sobre un nuevo tema conceptual que se está imponiendo de forma muy extensa entre nuestra comunidad profesional. Nos referimos a la denominada *biopsia líquida*, una definición que a primera vista resulta algo confusa.

El término biopsia líquida está siendo utilizado preferentemente en el diagnóstico oncológico y ha sido popularizado y difundido por las compañías de diagnóstico *in vitro*. Ahora bien, estas pruebas analizan las características del tumor a partir de una muestra de sangre en la que analizamos el ADN libre circulante. Aunque la procedencia de este ADN no se conoce con precisión, sabemos que tiene su origen en células tumorales y no tumorales que se necrosan o sufren procesos de apoptosis y liberan ADN al torrente circulatorio. En los pacientes oncológicos, aproximadamente el 50% del ADN circulante corresponde a ADN tumoral de pequeño tamaño [1]. En los cánceres avanzados podemos encontrar concentraciones de hasta 0.2 µg/mL. ¿Por qué la biopsia líquida ha levantado y está levantando tantas expectativas? La biopsia convencional es invasiva, la disponibilidad de tejido es limitada o en ocasiones inaccesible y a veces no somos capaces de capturar la heterogeneidad tumoral. Frente a esto, las ventajas que ofrece la biopsia liquida son evidentes: es mínimamente invasiva, menos costosa, es un mejor indicador de la heterogeneidad tumoral y ocasionalmente, permite monitorizar la respuesta y/o la resistencia al tratamiento [2]. Sin embargo, y aunque resulte evidente, es conveniente poner de manifiesto que la biopsia líquida no es más que una muestra sanguínea, como cualquier otra de las que se procesan en los laboratorios clinicos.

La biopsia líquida se utiliza principalmente en el diagnóstico de enfermedades oncológicas y se realiza a partir de ADN libre circulante procedente de células tumorales. En este ADN podemos detectar alteraciones puntuales, translocaciones o incluso alteraciones en la metilación. Sin embargo, hemos de hacer notar que esta metodología no la utilizamos únicamente en el ámbito estrictamente oncológico. Probablemente en el diagnóstico prenatal no invasivo también puede tener una gran trascendencia desde el punto de vista sanitario y social. Podemos detectar ADN libre circulante fetal en sangre periférica desde el primer trimestre de embarazo, convirtiéndose de esta forma en una técnica muy potente de diagnóstico prenatal [3], [4]. Además está exenta de los inconvenientes que representa la obtención de líquido amniótico o vellosidades coriales.

El análisis del ADN libre circulante con finalidad diagnóstica en el ámbito oncológico no está exento de complejidad y esto resulta fácilmente comprensible si examinamos los determinantes celulares de transformación tumoral. La actividad de los oncogenes y genes supresores de tumores establece si una célula concreta se transforma en cancerosa. El desequilibrio entre estos dos factores dará lugar a un crecimiento celular incontrolado, inhibición de la apoptosis, inducción de angiogénesis y, en última instancia, invasión celular y metástasis. Si hablamos de oncogenes podemos establecer tres categorías principales: los receptores tirosina quinasa, los factores de crecimiento y los factores de activación transcripcional. Por otro lado, los genes supresores de tumores pueden ser reguladores del ciclo celular o inhibidores de la proliferación.

Los oncogenes se encuentran en muchas vías de señalización celular que están controladas por los receptores tirosina quinasa. Estos son receptores de membrana que al interactuar con factores de crecimiento, citoquinas u hormonas activan la vía de señalización correspondiente. Ejemplos de receptores tirosina quinasa son el receptor del factor de crecimiento epitelial, el receptor del factor de crecimiento vascular o el de fibroblastos. Una de las vías de señalización más universalmente conservada en las células eucariotas es la vía Ras-Raf-Mek-Erk. En presencia de un agonista específico, como un factor de crecimiento, el receptor tirosina quinasa envía una señal de fosforilación activando la vía y dando lugar a diferentes respuestas biológicas como crecimiento, supervivencia y proliferación o diferenciación celular. Muchas de las dianas analíticas que analizamos en la biopsia líquida se centran en las anomalías génicas que regulan estos mecanismos.

Además, de saber si las mutaciones que podemos detectar se corresponden con un oncogen o un gen supresor de tumores, también existen diferentes situaciones en función del tipo de alteración portadora de la mutación. Así podemos encontrar variaciones en un único nucleótido, inserciones o deleciones genómicas, fusiones y variaciones en el número de copias. Con todo esto únicamente se trata de subrayar la extraordinaria complejidad que puede adquirir el diagnóstico molecular en las enfermedades oncológicas. Además debemos considerar que la patología oncológica es un proceso dinámico y que nosotros podemos estar examinando su situación en fases diferentes de su evolución: cuando se producen los primeros cambios neoplásicos, en circunstancias de heterogeneidad tumoral, o cuando estamos en fase de tratamiento y tengamos respuesta con remisión o en fase de recurrencia. Estas diferentes situaciones también se pueden ver reflejadas en cambios en el ADN libre circulante. No resulta sorprendente que ante todo este alud de información, se haya producido una cierta prevención a la hora de acoger este tipo de técnicas en el laboratorio clínico más convencional. En cualquier caso, aquellos lectores interesados en profundizar en estos conceptos disponen de una excelente revisión de Maria Arechederra y cols. en este mismo número de *Advances in Laboratory Medicine* [5].

Llegados a este punto me gustaría atraer la atención del lector sobre las principales estrategias analíticas y de instrumentación que tenemos a nuestro alcance. Esencialmente podemos hablar de dos estrategias diferenciadas. Una la utilizaremos cuando tenemos bien definidas las mutaciones que queremos evaluar. Es una estrategia que llamamos dirigida y que consiste en el uso de PCR en tiempo real en sistemas múltiplex que puede estar asociada o no, a secuenciación posterior y con el correspondiente análisis bioinformático. Lo que se debe subrayar es que en estos momentos disponemos de instrumentación específica para esta tecnología y de los *kits* correspondientes. La segunda estrategia está basada en la técnica denominada *Next Generation Sequencing*. En este caso detectamos mutaciones conocidas y no conocidas, siguiendo una estrategia diagnostica no dirigida. Aquí, por lo tanto no dispondremos de *kits* específicos para determinadas mutaciones y el flujo de trabajo se inicia con un extractor automático de ADN, seguido por sistemas manuales o automatizados de preparación de librerías, dependiendo de la cantidad de muestras, y sistema de ultra secuenciación [6]. Naturalmente, también necesitaremos del análisis bioinformático correspondiente que puede incluir o no, desarrollos *in house* [7]. Asimismo debemos mencionar los sistemas desarrollados para aplicaciones específicas como por ejemplo, el cribado poblacional en mujeres embarazadas durante el primer trimestre de gestación. Este consiste en el análisis del ADN fetal libre circulante en la sangre materna [4]. Lo importante a destacar es que en estos momentos disponemos de alternativas tecnológicas diversas, que se adaptan a necesidades diagnósticas diferentes y que además presentan un elevado componente de automatización que muy probablemente se verá incrementado en los próximos años.

Otro aspecto muy crucial a considerar es que el impacto económico que el diagnóstico molecular tendrá en nuestros laboratorios será muy importante. Las previsiones para los próximos diez año en los EE.UU. son que el número de test se incrementará en 10 veces aproximadamente [8]. Dos razones principales justifican esta afirmación: el diagnóstico molecular está facilitando el uso de terapias selectivas que han demostrado eficacia clínica y además, las aplicaciones de supervisión y control de tratamiento experimentarán gran crecimiento.

Estas previsiones sugieren de forma muy consistente que la biopsia liquida se convertirá en una actividad muy relevante en los laboratorios clínicos. Esta afirmación cobra mucha más fuerza si tenemos en cuenta las aplicaciones no oncológicas del ADN libre circulante. De hecho, existen numerosos estudios publicados estos últimos años que exploran la utilidad diagnóstica del ADN libre circulante en diversas patologías. Así se ha descrito su utilidad en el infarto de miocardio, el ictus o como biomarcador de rechazo en el trasplante, También como factor pronóstico en el trasplante de islotes pancreáticos [9], [10].

Para finalizar quisiera mencionar la fase preanalítica. Como es bien sabido la obtención de resultados fiables depende de una preanalítica cuidadosa. Y, como era de esperar esto también se produce al determinar el ADN libre circulante. En este sentido, un estudio multicéntrico europeo en el que se investigó el riesgo de cáncer en función de la concentración de ADN demostró la existencia de grandes diferencias intercentro en relación a este último parámetro [11]. Este estudio demuestra que, de la misma forma que sucede con cualquier otro parámetro de laboratorio, las condiciones preanalíticas constituyen un factor crítico para obtener información fiable de la biopsia líquida.

En conclusión, la información actualmente existente, así como la prospectiva de futuro indican que la incorporación de los procedimientos relacionados con la biopsia liquida/ADN libre circulante a los laboratorios de diagnóstico clínico constituye un reto profesional que deberemos afrontar en el inmediato futuro.
